# Active Case Finding of Tuberculosis (TB) in an Emergency Room in a Region with High Prevalence of TB in Brazil

**DOI:** 10.1371/journal.pone.0107576

**Published:** 2014-09-11

**Authors:** Denise Rossato Silva, Alice Mânica Müller, Karina da Silva Tomasini, Paulo de Tarso Roth Dalcin, Jonathan E. Golub, Marcus Barreto Conde

**Affiliations:** 1 Pulmonology Department, Faculdade de Medicina da Universidade Federal do Rio Grande do Sul – Hospital de Clínicas de Porto Alegre, Porto Alegre, Brazil; 2 Programa de Pós-Graduação em Ciências Pneumológicas, Universidade Federal do Rio Grande do Sul, Porto Alegre, Brazil; 3 Epidemiology Department, Johns Hopkins School of Medicine, Baltimore, Maryland, United States of America; 4 Instituto de Doenças do Tórax, Universidade Federal do Rio de Janeiro, Rio de Janeiro, Brazil; Kliniken der Stadt Köln gGmbH, Germany

## Abstract

**Setting:**

Public hospital emergency room (ER) in Porto Alegre, Brazil, a setting with high prevalence of tuberculosis (TB) and human immunodeficiency virus (HIV) infection.

**Objective:**

To determine the prevalence of PTB, using a symptom based active case finding (ACF) strategy in the ER of a public hospital in an area with high prevalence of TB and HIV, as well as variables associated with pulmonary TB diagnosis.

**Methods:**

Cross sectional study. All patients ≥18 years seeking care at the ER were screened for respiratory symptoms and those with cough ≥2 weeks were invited to provide a chest radiograph and two unsupervised samples of sputum for acid-fast bacilli smear and culture.

**Results:**

Among 31,267 admissions, 6,273 (20.1%) reported respiratory symptoms; 197 reported cough ≥2 weeks, of which pulmonary TB was diagnosed in 30. In multivariate analysis, the variables associated with a pulmonary tuberculosis diagnosis were: age (OR 0.94, 95% CI: 0.92–0.97; p<0.0001), sputum production (OR 0.18, 95% CI 0.06–0.56; p = 0.003), and radiographic findings typical of TB (OR 12.11, 95% CI 4.45–32.93; p<0.0001).

**Conclusions:**

This study identified a high prevalence of pulmonary TB among patients who sought care at the emergency department of a tertiary hospital, emphasizing the importance of regular screening of all comers for active TB in this setting.

## Introduction

Actively screening for tuberculosis (TB) is now recommended in high incidence settings in response to the slow decline in global TB incidence [1], [2]. Brazil has a moderate TB epidemic (TB cases in 2010, 36/100,000 inhabitants) [3]. However, some cities, such as Porto Alegre in southern Brazil, continue to have very high rates (100 cases/100,000 inhabitants/year) [4]. Brazil’s current policy states that patients admitted in emergency rooms should be screened for TB and suspects should be isolated until the results of sputum smear microscopy are known [5]. However, this policy is rarely followed. Thus, the aim of this study was to determine the prevalence of PTB, using a symptom based active case finding (ACF) strategy, in an emergency room of a public hospital in Porto Alegre city (Brazil), a setting with the highest TB incidence in Brazil.

## Materials and Methods

### Study design and setting

We conducted a cross-sectional study at the ER of Hospital de Clínicas de Porto Alegre, a tertiary care, university-affiliated hospital with 750 beds, located in the city of Porto Alegre, Rio Grande do Sul State, in southern Brazil. The hospital admits approximately 170 active TB patients annually, and the primary risk factor for TB in this setting is HIV infection [6]. The study was approved by the Ethics Committee at Hospital de Clínicas de Porto Alegre in October 1^st^, 2009.

Between November 1^st^ 2009 and March 31^st^ 2011 all individuals aged 18 years or older attending the ER for any reason between 8∶00 AM and 6∶00 PM from Monday thru Friday were interviewed for respiratory symptoms (cough, dyspnea or chest pain) and all individuals reporting cough ≥2 weeks were invited to participate in the study. To ensure that all individuals visiting the ER were screened, the team was positioned at the registration counter at the main entrance of the ER. After signing informed written consent enrolled subjects were interviewed, underwent a physical examination and a chest radiograph (CXR). Patients were also instructed to provide two unsupervised sputum samples that day. Patients were excluded from the study if they did not complete the interview or did not provide at least one sputum specimen (spontaneous, induced or with bronchoscopy). Sputum specimens were stained with Ziehl-Neelsen (ZN) and cultured in Löwenstein-Jensen (LJ) medium following standard protocols at the Mycobacteria Laboratory at Hospital de Clínicas de Porto Alegre. All specimens that were culture-positive for mycobacteria were speciated to distinguish *Mycobacterium tuberculosis* from other non-tuberculous mycobacteria. Induced sputum was performed when patient had no spontaneous sputum production. Those with negative induced-sputum results still suspected with TB are then referred for bronchoscopy. Pulmonary TB (PTB) was diagnosed according to criteria established by World Health Organization, so that a patient with one positive AFB smear is considered a definite case. Smear negative and culture positive patients were also considered TB cases [7].

### Data Collection

Data collection instruments were pre-tested, validated and modified during a pilot study. In addition to demographic data (sex, age, and years of schooling), we surveyed participants about their history of smoking, alcohol use, drug use, co-morbidities and TB. A current smoker was defined as reporting smoking at least 100 cigarettes in their lifetime, and at the time of the survey were smoking at least one day a week. A former smoker was defined as reporting smoking at least 100 cigarettes in their lifetime but who, at the time of the survey, did not smoke at all. Never smoked reported having smoked <100 cigarettes in their lifetime. Alcohol abuse was defined as daily consumption of at least 30 grams (equivalent to a pint and a half of 4% beer) for men and 24 grams (equivalent to a 175 ml glass of wine) for women. Patients reported comorbidities including HIV infection, diabetes and cancer), and if they lived in a prison, shelter, or nursing home in the last 3 years. Patients with unknown HIV status were tested at the discretion of the ER physician. For that reason, we did not include HIV in the analysis.

An independent physician analyzed the CXRs and classified them as normal, suggestive of active TB, suggestive of inactive (healed) TB or abnormal but not suggestive of TB, according to previously described guidelines [8].

### Statistical Analysis

Data analysis was performed using SPSS 18.0 (Statistical Package for the Social Sciences, Chicago, Illinois). Data were presented as number of cases, mean ± standard deviation (SD), or median with interquartile range (IQR). Categorical comparisons between pulmonary TB and other respiratory diseases (ORD) groups were performed by chi-square test using Yates’s correction if indicated or by Fisher’s exact test. Continuous variables were compared using the independent samples *t*-test or Mann-Whitney test. Multivariate logistic regression analysis evaluated factors associated with PTB diagnosis, using selection of factors associated (p≤0.10) with PTB diagnosis in univariate analysis, controlled by sex and age. Hierarchical logistic regression models with predictors added one at a time were also examined to evaluate the possible collinearity among the predictors. The goodness-of-fit of the multiple logistic regression models was assessed using the Hosmer-Lemeshow test. Odds ratios (ORs) and nominal 95% confidence intervals (CIs) were presented. A two-sided p-value<0.05 was considered significant for all analyses.

## Results

**Figure 1:**
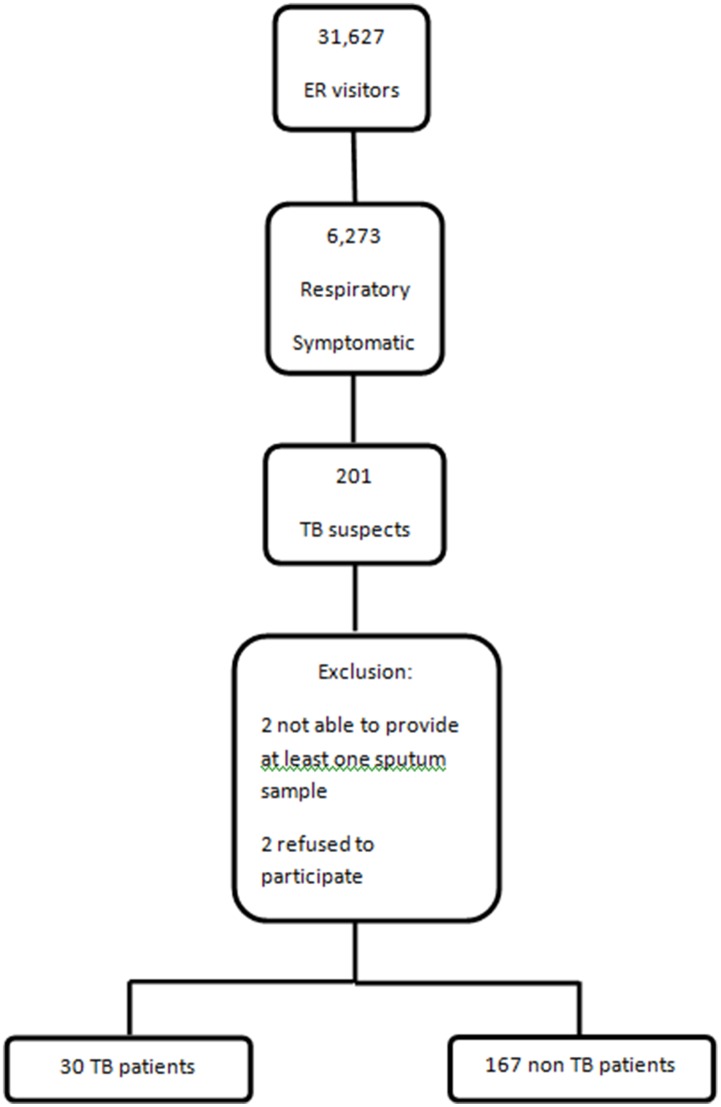
Study flow chart.

During the study period, 31,267 patients were seen in the emergency room, of which 6,273 (20.1%) reported respiratory symptoms (Figure 1). Among these 6,273 patients, 201 (3.2%) reported cough ≥2 weeks and were invited to participate in the study. Two patients refused to sign the consent form, 2 patients were not able to provide a sputum specimen, thus 197 subjects were enrolled in the study. Pulmonary TB was diagnosed in 15% (30/197), pneumonia in 23% (45/197), unspecified respiratory tract infection in 21% (41/197), chronic obstructive pulmonary disease (COPD) exacerbation in 14% (27/197), lung cancer 6% (11/197) and 43 subjects were diagnosed with unspecific diagnoses including sinusitis, asthma, pulmonary abscess, bronchiectasis, lymphoma, histoplasmosis, and pneumocystocis.

Among the 30 pulmonary TB patients, 21 (70%) were able to provide spontaneous sputum, 5 (16.7%) underwent sputum induction, and 4 (13.3%) bronchoalveolar lavage. There were 18 (60.0%) patients with smear positive results (17 among those with spontaneous sputum and one among those with who underwent induced sputum). Three patients were smear negative and culture positive. Among non-PTB patients, 132 (79.0%) were able to provide spontaneous sputum, 13 (7.8%) underwent sputum induction, and 22 (13.2%) bronchoalveolar lavage.

Among the 30 PTB cases, 19 (63.3%) reported cough for more than three weeks. Among the 27 patients who received an HIV test or knew their results, 12 (44%) were seropositive, and 8 of 12 co-infected patients reported cough for more than 3 weeks. The median duration of cough was 30 days (15–60 days) in PTB patients and 21 days (14–60 days) in non-PTB patients (p = 0.053). Non-PTB patients were older (mean age 56.4±17.6 years) than TB patients (mean age 41.3±14.7 years) (p<0.0001). Sputum production and dyspnea were more common in non-PTB patients as compared with PTB patients (153 [90.5%] vs 21 [70.0%], p = 0.002 and 135 [79.9%] vs 15 [50.0%], p<0.0001, respectively). Fever was more common in PTB patients (26 [86.7%]) than in non-PTB patients (88 [52.1%]) (p<0.0001). Radiographic findings typical of TB were more common in PTB patients (n = 17 [56.7%]) than in non-PTB patients (n = 19 [11.2%]) (p<0.0001) (Table 1).

**Table 1:** Characteristics of the sample.

Characteristics	Pulmonary TB	Other respiratory diseases	p value (univariated analysis)
	n = 30 (15.0%)	n = 169 (84.9%)	
**Demographic characteristics**			
Age, yr	41.3±14.7	56.4±17.6	<0.0001
Male sex	12 (40.0)	85 (50.3)	0.298
White race	19 (63.3)	115 (68.0)	0.612
**Current smoker**	8 (26.7)	44 (26.0)	0.942
**Alcoholism**	4 (13.3)	14 (8.3)	0.374
**Drug use**	1 (3.3)	8 (4.7)	0.734
**Institutionalization**	2 (6.7)	9 (5.3)	0.767
**Diabetes**	2 (6.7)	20 (11.8)	0.406
**Previous TB**	3 (10.0)	32 (18.9)	0.236
**Symptoms**			
Spontaneous sputum	21 (70.0)	153 (90.5)	0.002
Weight loss	24 (80.0)	104 (61.5)	0.052
Night sweats	20 (66.7)	87 (51.5)	0.124
Dyspnea	15 (50.0)	135 (79.9)	<0.0001
Chest pain	19 (63.3)	117 (69.2)	0.522
Fever	26 (86.7)	88 (52.1)	<0.0001
Hemoptysis	3 (10.0)	36 (21.3)	0.318
**Duration of cough** **before admission, days**	30.0 (15.0–60.0)	21.0 (14.0–60.0)	0.053
**Surgical mask use**	13 (43.3)	40 (23.7)	0.025
**Radiographic patterns**			
Typical of TB	17 (56.7)	19 (11.2)	<0.0001
Compatible with TB	13 (43.3)	65 (38.5)	0.614
Atypical	0 (0)	85 (50.3)	<0.0001
**Mechanical ventilation**	4 (13.3)	3 (1.8)	0.002
**Emergency room length of stay, d**	1.0 (0.5–2.0)	1.0 (0.5–3.0)	0.323
**Total length of stay, d**	9.5 (2.0–18.3)	2.0 (0.5–8.0)	0.001
**In-hospital mortality**	2 (6.7)	8 (4.7)	0.655

Continuous variables (age) are presented as mean ± SD; other data are presented as n/N (%): number of cases with characteristic/total number of cases (percentage in the group), or median (interquartile range).

Multivariate logistic regression revealed older age (OR 0.94, 95% CI: 0.92–0.97; p<0.0001) and sputum production (OR 0.18, 95% CI 0.06–0.56; p = 0.003) to be protective of developing TB, while radiographic findings typical of TB (OR 12.11, 95% CI 4.45–32.93; p<0.0001) markedly increased risk of TB.

## Discussion

We found that over 20% of people presenting at a large emergency department in Brazil for any cause, reported respiratory symptoms, that 3% of these patients were considered TB suspects based on the criteria of a cough for greater than or equal to 2 weeks, and that 15% of suspects had pulmonary TB. A previous study in Brazil [9] has reported a 5.9% prevalence of respiratory symptoms in emergency department. Another study in Rio de Janeiro, Brazil reported that 3% of TB suspects were ultimately diagnosed with TB [10]. However, the screening criteria in the Rio study was cough ≥1 week, thus significantly increasing the sample.

Current guidelines in Brazil state that patients with cough ≥3 weeks should be screened for TB [3]. In our sample, only 63% (19/30) of TB cases had cough for three weeks or more. This finding suggests that at least in the ER of our Hospital, we should consider for TB diagnosis those patients with cough lasting more than two weeks. In fact, a previous study in a primary care facility in Rio de Janeiro has already demonstrated that using cough ≥1 week as an eligibility criteria for passive case finding (PCF) of TB increased the detection of TB cases without a significant overload of the TB Lab [11].

ED serves as the frontline for patients with respiratory diseases in many developing and developed countries. Respiratory problems accounted for 5.9% to 16.2% of total emergency room visitations in previous studies [9], [12]. The majority of these patients were diagnosed with acute respiratory infections, like pneumonia and unspecified respiratory tract infection, as demonstrated in the present study. In a retrospective study [9] conducted in an emergency room of Nigeria, pneumonia was also the most common diagnosis, comprising 34.5% of cases.

Among symptomatic respiratory patients in our study, 15.1% were diagnosed with PTB. TB in ED is a major concern in other countries too [13]–[16]. Some studies [15], [16] were conducted to develop rapid decision instruments for isolation of patients at risk for PTB in high-PTB-prevalence populations. In a study [14] conducted in Edmonton, Canada, they found that ED was heavily utilized by urban tuberculosis patients pre-diagnosis. In a retrospective study [9] carried out on adult patients that presented in ED with respiratory complaints, PTB accounted for as much as 29.4% of cases. Nevertheless, this study was conducted in Nigeria, one of the highest TB burdens in the world (311 cases per 100,000).

The sensitivity of sputum smear microscopy in our study was similar to that reported in literature (60–70%) [5]. Acid-fast bacilli (AFB) smear has a low sensitivity among patients with noncavitary pulmonary TB and it is not able to provide any information about the resistance of the *Mycobacterium tuberculosis*. In addition, in an ER diagnosis and therapeutic decision should be as fast and accurate as possible. In this context, the Xpert *MTB/RIF* assay (GeneXpert) could play an important role. This is a fully automated molecular diagnostic test that can simultaneously detect *Mycobacterium tuberculosis* complex DNA and mutations associated with rifampicin resistance directly from sputum specimens in less than 2 hours, minimizing staff manipulation and biosafety risk [17]. The rapid diagnosis of TB in the ER has an impact on transmission dynamic of TB, since the ER is a high-risk site for potential propagation of the disease, with frequent lack of biosafety measures for the prevention of TB [18].

In spite of the number of complaints, sputum production was found to be a negative predictor of PTB. One plausible explanation for this finding is that patients who could expectorate were diagnosed at a primary health care level, and those without sputum production impose diagnostic difficulties. Actually, it is well known that patients who were unable to produce sputum or with negative sputum smear microscopy results have more frequently delayed diagnosis of PTB [19], [20]. In addition, induced sputum and bronchoscopy have been used to obtain specimens for diagnosis in these patients [21]–[23], but these procedures are not largely available in primary health care centers.

Dyspnea was another symptom found to be a negative predictor of PTB in our sample, at least in univariate analysis. This finding is in agreement with a retrospective study that identified variables associated with a PTB diagnosis in inpatients [24]. Other authors also found in outpatients that dyspnea was negatively associated with PTB [25]. On the other hand, fever was more common in patients with TB. This finding is probably explained by the fact that in non-TB group we have also patients with non-infectious diseases, that course with no fever.

Radiology plays an important role in the screening for TB. Abnormalities on chest radiograph typical of TB were associated with an increased risk of TB in our study. In a previous investigation, only consolidation was associated with the presence of TB [26]. Chest radiographs with characteristics considered typical of TB, such as upper lobe infiltrate or cavities are usually y reported clinical predictors of TB [21]–[23], [25].

It is difficult to explain the high number of TB cases diagnosed in the ER. It may reflect the failure of primary health care unit to detect and treat TB cases as well as the severity of disease [6], [26]–[27]. Maior et al (2012) recently reported that 70% of patients treated for TB in a primary health clinic in Nova Iguaçu city (Rio de Janeiro State, Brazil), a city with a TB incidence of 76 cases/100,000 population, initially sought medical attention at an emergency room (ER) rather than the primary health care clinic. This finding suggests a failure in the access and/or receptiveness in the primary health care system [10].

The present study has limitations. Although the prevalence of HIV infection among the TB patients in our sample (44%) has been four times greater the mean rate of co-infection in Brazil (10%), we could not evaluate if it was a predictor of PTB because patients were tested at the discretion of the ER physician. Also, the TB prevalence could be underestimated because only subjects ≥18 years old reporting cough ≥2 weeks were screened for TB.

In conclusion, this study emphasizes the need of ACF in ER of settings with high prevalence of TB and comorbidities like HIV. Studies on ERs localized in settings with different TB and HIV incidences are necessary to confirm our findings.
